# Digital Pixel Sensor Array with Logarithmic Delta-Sigma Architecture

**DOI:** 10.3390/s130810765

**Published:** 2013-08-16

**Authors:** Alireza Mahmoodi, Jing Li, Dileepan Joseph

**Affiliations:** Department of Electrical and Computer Engineering, University of Alberta, 9107 116 Street NW, Edmonton, AB T6G 2V4, Canada; E-Mails: mahmoodi@ualberta.ca (A.M.); jl11@ualberta.ca (J.L.)

**Keywords:** CMOS image sensors, dynamic range, digital pixel sensors, nonlinear response, signal-to-noise ratio, analog-to-digital conversion, delta-sigma modulation, decimation

## Abstract

Like the human eye, logarithmic image sensors achieve wide dynamic range easily at video rates, but, unlike the human eye, they suffer from low peak signal-to-noise-and-distortion ratios (PSNDRs). To improve the PSNDR, we propose integrating a delta-sigma analog-to-digital converter (ADC) in each pixel. An image sensor employing this architecture is designed, built and tested in 0.18 micron complementary metal-oxide-semiconductor (CMOS) technology. It achieves a PSNDR better than state-of-the-art logarithmic sensors and comparable to the human eye. As the approach concerns an array of many ADCs, we use a small-area low-power delta-sigma design. For scalability, each pixel has its own decimator. The prototype is compared to a variety of other image sensors, linear and nonlinear, from industry and academia.

## 1. Introduction

With image sensors, dynamic range (DR) is the ratio of largest non-saturating signal to smallest detectable signal. Leading researchers, such as El Gamal [1] and Yadid-Pecht [2], have called for wide DR solutions having good image quality. As Smith [3] explains, image quality may be measured by signal-to-noise-and-distortion ratio (SNDR), a function of temporal noise and residual fixed pattern noise (FPN) that is less than or equal to signal-to-noise ratio (SNR). Recently, after surveying 24 published image sensors relative to the human eye, Skorka and Joseph [4] have shown that achieving wide DR, *i.e.*, ≥ 126 dB, and high peak SNDR (PSNDR), *i.e.*, ≥ 36 dB, remains difficult at video rates.

This paper proposes a new architecture for image sensors to tackle the above difficulty In this architecture, a delta-sigma (ΔΣ) analog-to-digital converter (ADC) is integrated in each pixel of a logarithmic CMOS image sensor. The result is a nonlinear digital pixel sensor (DPS) array, as opposed to the typical active pixel sensor (APS) array, where response to light stimulus is linear and where ADCs are integrated at the column, chip or board level. The potential of this new architecture is demonstrated with experimental results from a prototype, which shows wide DR and high PSNDR at video rates.

Unlike linear APS arrays, logarithmic APS arrays achieve wide DR easily at video rates, but suffer from low PSNDR. The PSNDR is *not* low because of the well-known FPN of logarithmic arrays. Temporal noise *is* the limiting factor, as methods have been developed to correct FPN effectively [5]. Relative to signal, temporal noise is high, because logarithmic sensors are non-integrating and compressive—a wide DR of photocurrent is converted to a narrow DR of voltage continuously. By introducing pixel-level ADCs, as shown here, temporal noise and FPN at the column, chip and board level are excluded, because off-pixel readout is 100% digital. Furthermore, using a ΔΣ ADC, instead of a Nyquist-rate one, means in-pixel noise filtering is done robustly with digital signal processing (DSP).

Other researchers have worked on pixel-level ΔΣ ADCs. Whereas a ΔΣ ADC needs both modulation and decimation, decimation was done outside the pixel in all previous work, which limits frame rate and/or array size. Moreover, previous work tried to increase the DR of linear sensors. Fowler *et al.* [6] integrated a simplified modulator with a linear sensor; the resulting DPS suffered from high FPN [7]. McIlrath [8] integrated a pseudo-modulator with a linear sensor; wide DR was not achievable [9]. Joo *et al.* [10] integrated a true modulator with a linear sensor, but did not report DR. Walker *et al.* [11] integrated pixel-level modulators and column-level decimators with linear sensors, but also did not report DR. Recently, Ignjatovic *et al.* [12] integrated mostly pixel-level modulators (some parts were at the column level) and a board-level decimator with linear sensors; they reported a 74 dB intra-scene DR, 52 dB worse than that of the human eye [4].

When taken to the megapixel scale, DPS arrays require upwards of one million ADCs per chip. Therefore, considerable effort must be taken to reduce area usage and power consumption per ADC. In a conference proceeding [13], Mahmoodi and Joseph presented a method to design small-area low-power column-level ΔΣ modulators. In another conference proceeding [14], Mahmoodi and Joseph extended the method to design small-area low-power pixel-level ΔΣ ADCs, using patent-pending decimators [15]. These methods were presented only with simulated designs of ADCs-there were no sensors and no experiments. In contrast, this paper is about image sensors and, to experimentally validate the proposed architecture, a prototype image sensor was designed, built and tested.

While the proposed architecture approaches human eye performance in terms of DR and PSNDR at video rates, it remains limited in terms of dark limit (DL) and spatial resolution (SR). State-of-the-art image sensors also suffer from high DL, relative to the human eye, but their SR is competitive [4,16]. Fortunately, emerging methods for vertical integration are compatible with the proposed architecture, and these methods are expected to enable lower DL and higher SR [17].

A prototype of the proposed architecture is presented in Section 2. Through testing and analysis, Section 3 validates the proposed architecture. Section 4 discusses pros and cons of the approach and Section 5 summarizes contributions.

## 2. Prototype

**Figure 1.**
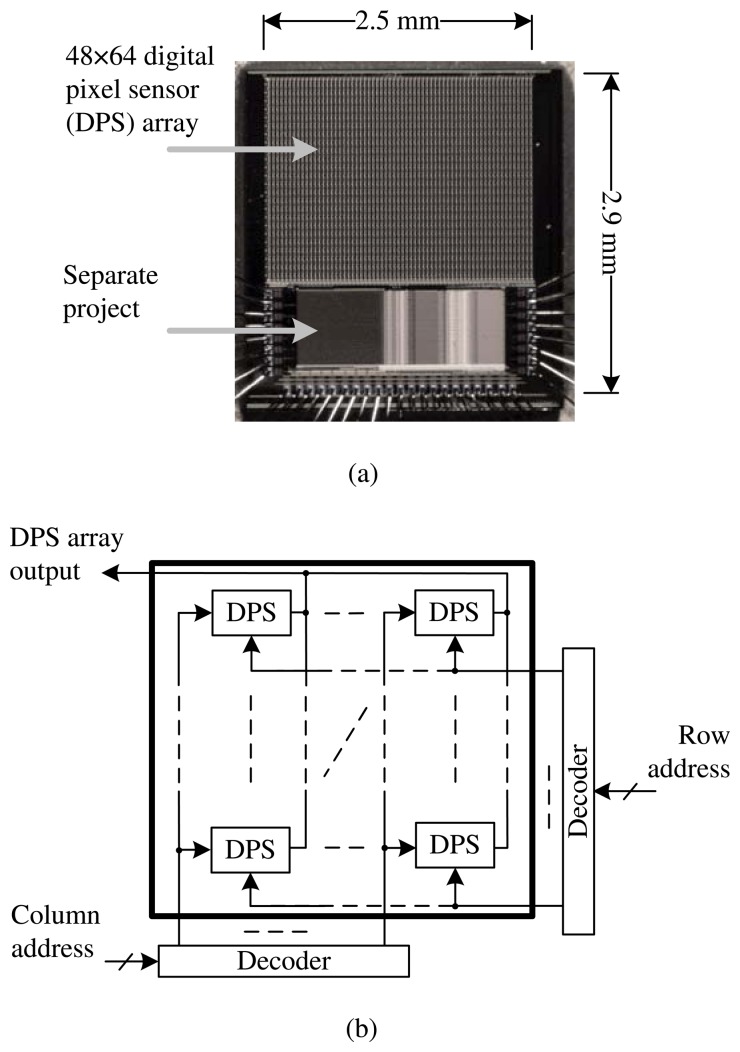
Prototype of proposed image sensor architecture. (a) Micrograph of chip, fabricated in 0.18 μm complementary metal-oxide-semiconductor (CMOS) technology; (b) floor plan of project, having a digital pixel sensor (DPS) array with logarithmic delta-sigma (ΔΣ) architecture. The array occupies 1.9 × 2.5 mm^2^ and uses 915, 456 transistors.

This paper concerns a new image sensor architecture, where ΔΣ ADCs are fully integrated, including decimators, at the pixel level with logarithmic, not linear, sensors. A prototype is required to validate the proposed architecture. Figure 1 gives the micrograph and floor plan of an image sensor design that was fabricated in 0.18 μm one-poly six-metal (1P6M) standard CMOS technology. Key circuit-and-system (CAS) details are discussed below, with reference to two conference proceedings [13,14] and one patent application [15], all published. Other details are available in a Ph.D. thesis [18].

To conserve resources, a small DPS array was designed, having 48 × 64 identical pixels. The goal was to achieve wide DR and high PSNDR, relative to the human eye, at video rates. While all pixels acquire signals in parallel, readout is done in sequence. Row and column decoders are used to address each pixel. The chip also has circuits for digital clocks and analog biases. Reference clocks and biases are supplied by a field-programmable gate array (FPGA) and a printed circuit board (PCB).

**Figure 2.**
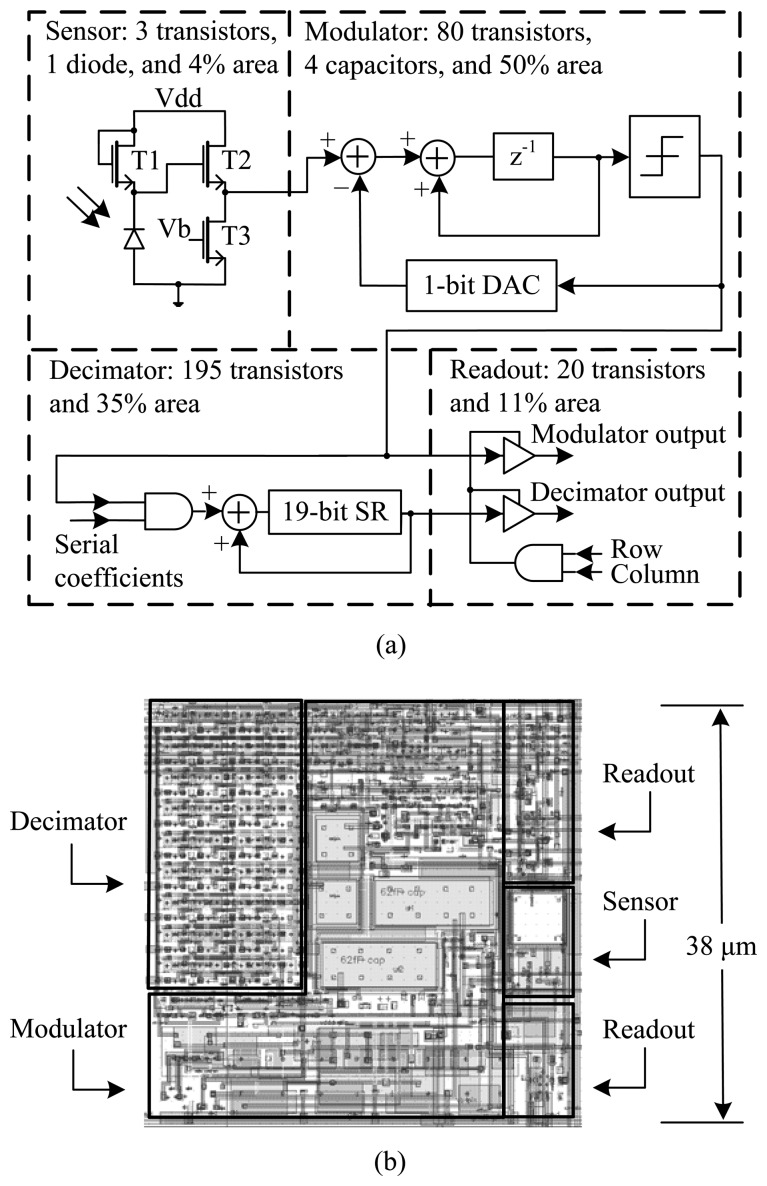
One DPS in the DPS array. (**a**) Schematic and (**b**) layout of one pixel, comprising a logarithmic sensor, a modulator, a decimator and a readout circuit. Here, “SR” and “DAC” mean “shift register” and “digital-to-analog converter,” respectively. The pixel occupies 38 × 38 μm^2^ and uses 298 transistors.

As shown in Figure 2a, each pixel of the image sensor has a logarithmic sensor, a first-order ΔΣ modulator, a bit-serial decimator and a readout circuit. In the logarithmic sensor, a reverse-biased diode turns input luminance into current. After a logarithmic current-to-voltage conversion by a subthreshold load, a source follower buffers the output voltage for data conversion. The ΔΣ ADC has a modulator and decimator in series, where the input and output clock are at *f*_mod_ and *f*_dec_, respectively. Normally, only the decimator output is read out. However, the modulator signal is available for debugging.

During data conversion, each modulator oversamples and quantizes, at *f*_mod_, the analog signal of the logarithmic sensor. Quantization noise produced by a comparator is shaped toward higher frequencies. The decimator low-pass filters the one-bit digital signal from the modulator and downsamples it to *f*_dec_, increasing the number of bits in the process. Bits are stored in a register, which has extra bits to handle noise. During data readout, each pixel is selected in turn, and decimator bits are outputted serially.

With DPS arrays, both area usage and power consumption per ADC are critical for megapixel scalability. Thus, a small-area low-power design, due to Mahmoodi and Joseph [13–15], is employed for the ADC. As shown in Table 1, detailed schematics are available from the academic and patent record; therefore, they are not repeated here. Scalability considerations are discussed in Section 4.

The SNDR of each DPS, where a logarithmic sensor is combined with a linear ADC, is limited by the peak SNR (PSNR) of its ADC. To realize 1% luminance accuracy, *i.e.*, 40 dB SNDR of the DPS, over five decades of luminance, *i.e.*, 100 dB DR of the DPS, the PSNR of the ADC must be at least:

(1) $$20\log_{10}(5\ln10/0.01)=61\;\text{dB}$$

given the logarithmic luminance-to-voltage conversion. This calculation does not account for offset variation of the logarithmic sensor with respect to the ADC reference voltages. Moreover, it does not account for the dependence of output quantization noise on input voltage [19].

Mahmoodi and Joseph [13,14] designed the ADC to support an oversampling ratio (OSR), *f*_mod_/*f*_dec_, of up to about 1,000. According to their Simulink and Cadence Spectre results, which mainly simulated quantization noise, this enabled the PSNR of the ADC to reach 80 dB, providing a sufficient margin to account for the aforementioned and other non-idealities. While this OSR may seem high, it is noteworthy that the oversampling rate is on the order of only 10 kHz with pixel-level data conversion.

In the first-order modulator, small capacitors are used with a differential-mode switched-capacitor integrator. There are two sampling capacitors, each 20 fF, and two integrating capacitors, each 60 fF. All four, visible in Figure 2b, are metal-insulator-metal capacitors that are laid out, “full custom,” with a capacitor-top-metal layer. Parasitic capacitances are about 1 fF, as extracted from the layout. Use of small capacitors has two implications, namely, higher *kT*/*C* noise and higher gain variation.

Mahmoodi and Joseph [13] modelled the *kT*/*C* noise in comparison to other noise sources and determined that the integrating capacitor should be at least 37 fF. To achieve a 1/3 gain, while meeting minimum-size rules, the 20 and 60 fF capacitors were chosen. The differential-mode voltage range at the comparator input is then 2·1/3 of the single-ended voltage range at the logarithmic sensor output. As Mahmoodi and Joseph explained, a first-order one-bit modulator is relatively insensitive to gain variation. There is only one integrator loop, and integrator output is passed only to a comparator.

A second-order comb filter, which uses a triangular impulse response, is commonly employed to decimate the output of a first-order modulator [19]. Because the impulse response duration is twice the Nyquist interval, implementing a compact decimator this way is difficult. Although a first-order comb filter has a simple implementation, its rectangular impulse response gives inadequate filtering.

Mahmoodi and Joseph [14,15] presented a compact yet effective decimator as follows. A one-stage bit-serial decimator is employed, using a parabolic impulse response of duration equal to the Nyquist interval. The filter coefficients, generated outside the DPS array, are broadcast to all pixels in parallel. Multiplication by the one-bit modulator signal is accomplished with an “and” gate, and accumulation is accomplished with a one-bit full adder and a multi-bit register. Through simulation, 10-bit coefficients and a 19-bit accumulator were chosen to meet the quantization noise specification.

Each frame period starts with parallel data conversion and ends with sequential data readout. The frame rate equals 1/(*T*_c_ + *T*_r_), where *T*_c_ and *T*_r_ are conversion and readout times, respectively. Here, *T*_c_ equals 1/*f*_dec_ or *OSR*/*f*_mod_. For the prototype image sensor, *f*_mod_ is fixed at 53 kHz, and the OSR is varied up to a maximum of 1,024. Readout takes a constant 14.5 ms, which is *T*_r_. Therefore, the frame rate is 30, 41 and 52 Hz for an OSR of 1,024, 512 and 256, respectively. Because the frame rate of standard television is 30 Hz, these are all video rates.

The image sensor plugs into a custom PCB, which connects, in turn, to an Altera Cyclone II FPGA board, from Bitwise Systems, that runs custom firmware. Digital inputs and outputs of the chip interface to the FPGA through buffers and level converters on the PCB. The FPGA board includes a QuickUSB module to communicate with a PC through a Universal Serial Bus (USB) port, which also powers the entire system. PCB and FPGA boards are mounted inside a custom camera body, into which a Fujinon CF25HA-1 lens screws.

In this manner, the image sensor prototype is configured and operated. Video frames are read by the PC in real time. Before or after real-time FPN correction in the PC, the video is displayed on a monitor and/or saved for further analysis.

## 3. Validation

This section validates the proposed architecture by presenting experiments done with a prototype. An overview of the testing is given first, followed by a detailed analysis. Comparison to the state of the art is done in Section 4.

### 3.1. Specifications

Important specifications of image sensors are technology, spatial and temporal dimensions, power consumption and signal and noise properties [4]. Spatial dimensions are given by array size, pixel size and detector size. Temporal dimensions are given by video rate and duty cycle, *i.e.*, the proportion of each frame period actually spent imaging. Power consumption may be decomposed into analog and digital parts. Signal and noise properties include PSNDR, PSNR, DL and DR.

For the proposed architecture, Table 2 gives specifications of the first prototype. Whereas the first six entries are controlled, the last six are measured. Power is readily measured. In the CMOS die, analog (sensor and modulator) and digital (decimator and readout) parts are powered from different pins. On the PCB, they are supplied by the same 1.8 V regulator. Small resistors are placed in series to measure average currents, which are then multiplied to the supply voltage.

**Figure 3.**
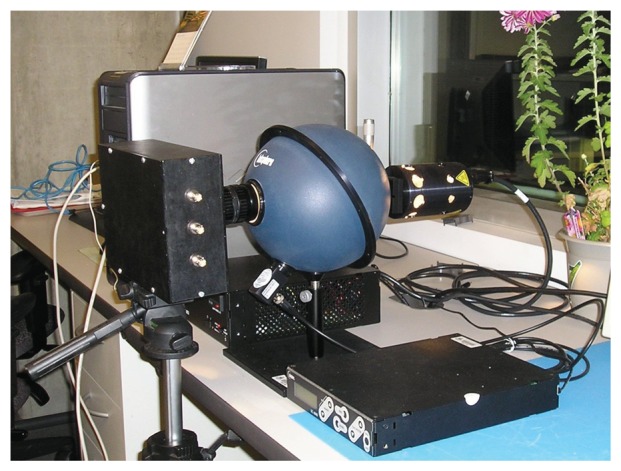
Test setup for the image sensor prototype. A printed circuit board (PCB) with the image sensor and a field-programmable gate array (FPGA) board are embedded in a camera body with a lens. This imaging system is affixed to an illuminated integrating sphere.

To measure signal and noise properties, a Labsphere USS-800C-100R was used, as shown in Figure 3. This continuous uniform source system includes a three-port integrating sphere, with a halogen lamp and a photopic detector connected to two ports. Through a regulated DC power supply, a constant luminance exits the third port, where the image sensor prototype with lens is placed. The lamp port includes a variable aperture, as does the camera lens. By varying both apertures, the image sensor is tested over a wide DR of luminance.

The measured data comprises *mnp* pixel responses, *y_ijk_*, where: *i* indexes luminance stimuli, *x_i_*, with 1 ≤ *i* ≤ *m*; *j* indexes pixels of the image sensor, with 1 ≤ *j* ≤ *n*; and *k* indexes consecutive frames, with 1 ≤ *k* ≤ *p*. For linear or nonlinear pixels, the input-output relationship is given by:

(2) $$y_{ijk}=f_{j}(x_{i})+\varepsilon_{ijk}$$

where *f_j_* varies with pixel, due to FPN, and *ε_ijk_* represents temporal noise and spatial distortion (residual FPN). FPN due to *f_j_* variation may be corrected after calibration.

An image sensor is an array of pixel sensors—in this case, a DPS array For each luminance, *x_i_*, and for each pixel, *j*, input-referred SNDR and SNR are defined as follows:

(3) $${\text{SNDR}}_{ij}=\frac{x_{i}}{\Delta x_{ij}^{nd}}=\frac{x_{i}}{\Delta y_{ij}^{nd}}|f_{j}^{\prime }(x_{i})|$$

(4) $${\text{SNR}}_{ij}=\frac{x_{i}}{\Delta x_{ij}^{n}}=\frac{x_{i}}{\Delta y_{ij}^{n}}|f_{j}^{\prime }(x_{i})|$$

where:

(5) $$\Delta y_{ij}^{\text{nd}}=\sqrt{\frac{1}{p}\overset{p}{\underset{k=1}{\text{∑}}}{\left(y_{ijk}-f_{j}(x_{i})\right)}^{2}}$$

(6) $$\Delta y_{ij}^{\text{n}}=\sqrt{\frac{1}{p-1}\overset{p}{\underset{k=1}{\text{∑}}}{\left(y_{ijk}-{\bar{y}}_{ij}\right)}^{2}}$$

are output-referred root-mean-square (RMS) values of noise-and-distortion and noise, respectively Denominators *p* and *p* – 1, in Equations (5) and (6) respectively, are the degrees of freedom. Means, *ȳ_ij_*, are computed by averaging *y_ijk_* over *k*, which represents time.

At each luminance, median SNDR and SNR of all pixels are taken as SNDR and SNR, respectively, of the array. Given smooth functions of luminance, maximum values of array SNDR and SNR are taken as PSNDR and PSNR, respectively. Finally, for the widest continuous interval of luminances, [*x*_min_, *x*_max_], over which array SNDR is always nonnegative, *x_min_* and *x*_max_/*x*_min_ are taken as DL and DR, respectively Because SNDR correlates with image quality better than SNR, it is preferable to use it for DL and DR estimation.

### 3.2. Characterization

The proposed image sensor architecture uses logarithmic pixels. Therefore, wide DR is expected at video rates. However, logarithmic sensors normally suffer from low PSNDR. Thus, it is important to characterize the noise and distortion realized by the image sensor prototype, which integrates ΔΣ ADCs into logarithmic pixels for the first time.

Using the test setup shown in Figure 3, images were taken at 22 different luminances. All 48 × 64 pixels worked, and 50 consecutive frames were captured at 30 fps for each luminance. As it was affected by system initialization, the first frame was later discarded. Thus, for the indexed data, *y_ijk_*, of Equation (2), index dimensions *m*, *n* and *p* are 22, 3,072 and 49, respectively. The experiment was repeated at 41 and 52 fps.

**Figure 4.**
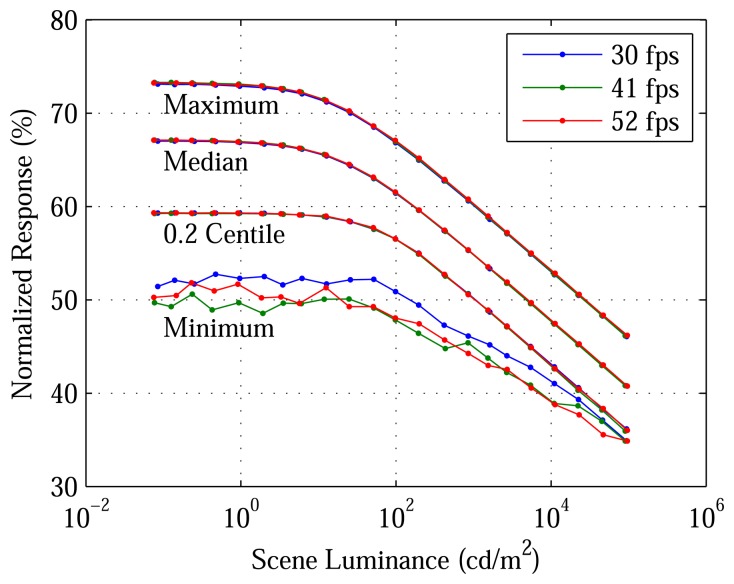
Time-average responses of the DPS array. While luminance inputs are uniform, pixel outputs vary due to fixed pattern noise (FPN). For 0.2% of pixels, ADC nonlinearity contributes significantly to FPN. For 99.8% of pixels, however, responses are simple logarithmic functions of stimuli, and FPN is readily corrected.

Figure 4 plots, *versus* luminance, selected time averages, *ȳ_ij_*, divided by 43,691, 5,430 and 659 LSB (least significant bits), respectively, at 30, 41 and 52 fps. Normalization is employed, because the maximum ADC output depends on OSR, although the minimum one is always 0 LSB. ΔΣ ADCs and logarithmic sensors have a synergy, which has been exploited. The former offer high precision and accuracy over the low voltage range of the latter. In this 1.8 V technology, the ADCs can cover up to a 0.7 V range, although only a smaller range is needed.

For the measured data, the responses, *y_ijk_*, depend on the stimuli, *x_i_*, according to Equation (2), with the following pixel model [5]:

(7) $$f_{j}(x_{i})=a_{j}+b_{j}\ln(c_{j}+x_{i})$$

where *a_j_*, *b_j_* and *c_j_* are called the offsets, gains and biases, respectively. Parameter variation causes FPN, which may be corrected in real time after one-time calibration. Here, FPN calibration is done using the time averages, *ȳ_ij_*. Linear pixels also suffer from offset and gain variation [1], called dark signal and photo response non-uniformity, respectively.

**Figure 5.**
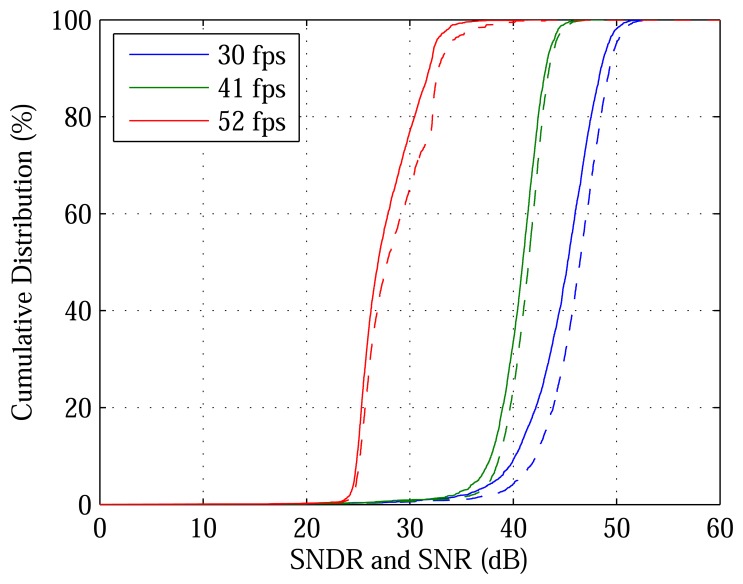
Cumulative distribution function (CDF) of SNDR and SNR at 23,000 cd/m^2^. SNR varies from pixel to pixel and increases with decreasing video rate, *i.e.*, increasing oversampling ratio (OSR). SNDR (solid line) is a little less than SNR (dashed line) at each video rate.

Figure 5 plots the cumulative distribution function (CDF), at one luminance and three video rates, of SNDR and SNR, across the DPS array. For example, the plots show that, at 23,000 cd/m^2^ and 30 fps, 97.5% of pixels have SNDR greater than 36 dB, which is the PSNDR of the human eye [4]. An advantage of the proposed architecture is that video rate may be traded with SNDR. Using a lower OSR, a higher video rate is achieved at the cost of lower SNDR. Video rate may also be increased by speeding up DPS readout. Notwithstanding the 14.5 ms taken to read out a frame, the DPS array can achieve the reported performance at 52, 104 and 207 fps, much faster than the reported 30, 41 and 52 fps, respectively.

**Figure 6.**
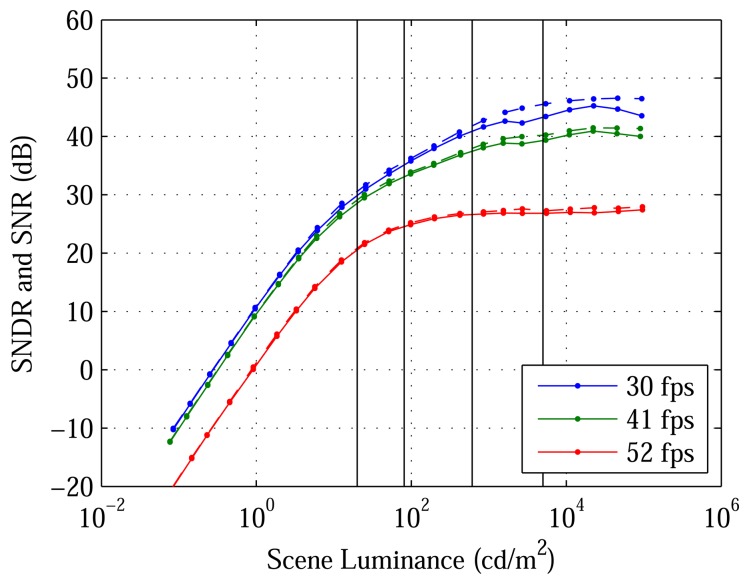
Median SNDR and SNR of the DPS array. Closeness of SNDR to SNR means that temporal noise, not residual FPN, is the limiting factor. SNDR peaks at about 23,000 cd/m^2^. Vertical lines mark highlights in Figure 7.

Figure 6 plots median SNDR and SNR *versus* luminance. The PSNDR, PSNR, DL and DR reported in Table 2 are taken from the 30 fps experiment, which corresponds to the maximum OSR (greatest noise filtering). At 23,000 cd/m^2^, median SNDR and SNR peak at 45 and 46 dB, respectively. Below 0.28 cd/m^2^, the DL, median SNDR is negative. Above 94,000 cd/m^2^, the bright limit (BL) of the test setup, median SNDR is unknown. Therefore, the DR is at least 110 dB.

In addition to quantitative analysis with uniform scenes, images were taken of non-uniform scenes for qualitative analysis. As with conventional image sensors, median filtering is used, after FPN correction, to eliminate “dead” (outlier) pixels. Five and three-pixel neighbourhoods are used for the interior and border, respectively. Finally, the PC maps linearized responses, from the FPN correction, to display tones using the sRGB standard [20]. Given a white point, whiter tones are saturated before scaling, gamma correction and rounding.

**Figure 7.**
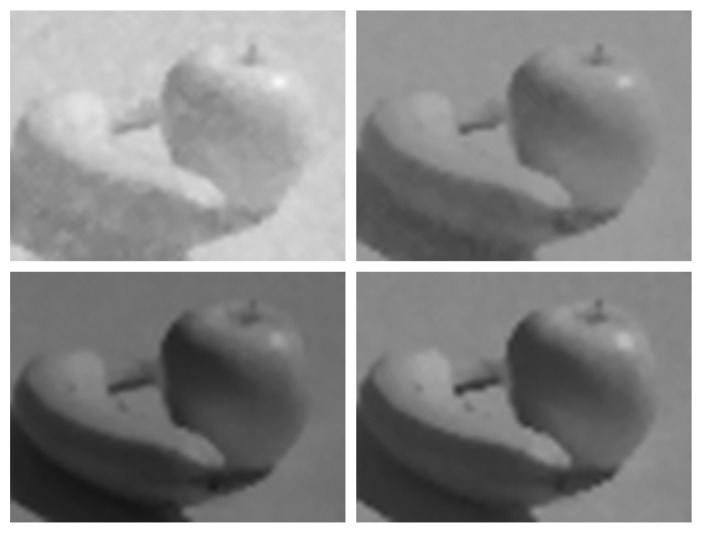
Images of a non-uniform scene taken at 30 fps. Four lens apertures are used to vary effective luminances of scene features. Tones are mapped for display using the sRGB standard [20]. Clockwise from top left, white points are 20, 81, 610 and 5,000 cd/m^2^, which correspond to apple highlights.

Figure 7 shows sRGB images of a scene captured with the DPS array prototype. By varying the lens aperture, the scene is captured at four optical gains spanning 48 dB. As luminances across the scene span 38 dB, the total DR of this experiment is 86 dB. The luminance of a feature in each image, noted in Figure 7, is cross-referenced in Figure 6. At lower luminance, SNDR is lower, and therefore, image quality is worse. At higher luminance, SNDR is higher, and therefore, image quality is better. In general, a feature in Figure 7 looks good if its SNDR is at least 36 dB, the PSNDR of the human eye [4].

## 4. Discussion

This section discusses the significance of the proposed architecture by comparing the performance of its first prototype, described above, to the human eye and other image sensors, using the method and data of a recent publication coauthored by the same principal investigator of this work.

### 4.1. Human Eye

Table 3 compares the image sensor prototype, with lens, to the human eye. By reviewing and analyzing independent literature, Skorka and Joseph [4] have specified the performance of the human eye in terms of the selected parameters. They also describe how to compute the same parameters for an image sensor, using either an ideal or real lens. Performance relative to the human eye is consistently given in decibels, where a positive value represents superiority.

For the prototype, most parameters in Table 3 are easily computed from Table 2. For example, temporal resolution (TR) is approximately half the video rate, according to the Nyquist theorem. Because they are referred to the scene, visual field (VF, in steradians) and SR (in cycles per degree) depend on focal length, ℓ. The equivalent focal length of the human eye, which is 17 mm, is used. With the actual 25 mm focal length, VF and SR are 0.0071 sr and 5.7 cpd, respectively.

The VF is computed as follows [4], from the solid angle, Ω_VF_, subtended by a right rectangular pyramid:

(8) $$\Omega_{\text{VF}}=4\arctan(\frac{wh}{2\ell \sqrt{4\ell^{2}+w^{2}+h^{2}}})$$

Here, *w* and *h* refer to the width (64 × 38 μm) and height (48 × 38 μm), respectively, of the sensor array, which forms the base of a pyramid. At the apex, the focal point sees the same solid angle looking toward the array or the scene.

Denoted *f*_SR_, the SR is computed as follows, from the focal-plane distance, *d*, subtended by one scene degree [16]:

(9) $$f_{\text{SR}}=d\cdot 0.5/p$$

(10) d=2ℓtan(0.5°)

Here, *p* is the pixel pitch (38 μm) and 0.5/*p* is the spatial Nyquist sampling rate. A more accurate definition requires the modulation transfer function (MTF) [4], which is difficult to compute unless aliasing is incorrectly ignored.

Performance trade offs are accepted practice in image sensor design. The significance of comparing to the human eye is: (1) the latter is a popular imaging system; (2) it achieves the stated specifications without compromise; and (3) the specifications are justified by natural selection. To discuss how the proposed architecture can help to rival the human eye's performance, it is necessary to consider other image sensors.

### 4.2. Other Image Sensors

**Figure 8.**
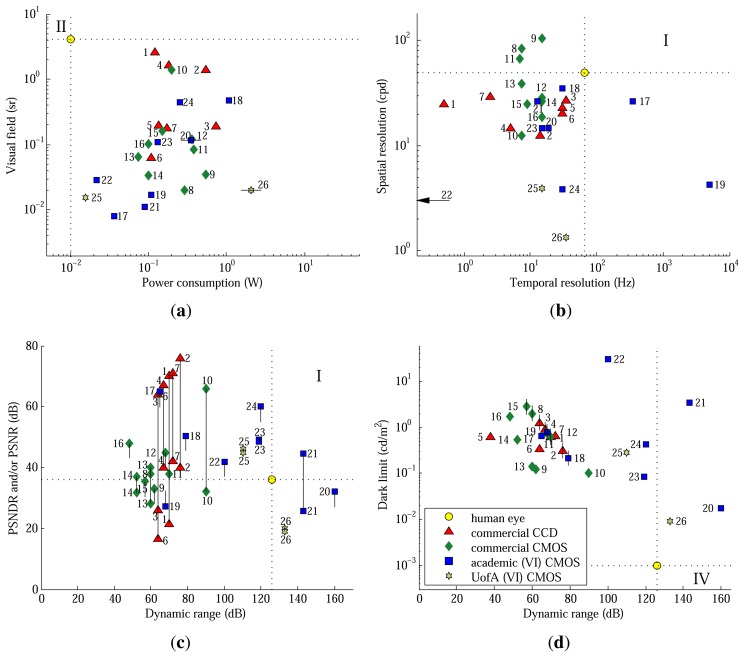
Imaging performance relative to other image sensors, adapted from Skorka *et al.* [4,16]. (**a**) Visual field *versus* power consumption; (**b**) spatial *versus* temporal resolution; (**c**) peak SNDR and/or SNR *versus* dynamic range; and (**d**) dark limit *versus* dynamic range. In the marked quadrants, performance exceeds the human eye on both parameters. Skorka *et al.* review designs 1–24. Designs 25 and 26 are from the University of Alberta, where the former is this work and the latter is by Skorka and Joseph [17].

Figure 8 compares the prototype to a variety of other image sensors, based on charge coupled device (CCD), CMOS and vertically-integrated (VI) CMOS technology. Sample calculations and a data table, which includes citations to datasheets and publications, are given by Skorka *et al.* [4,16] for designs 1–24, which were chosen based on documentation and diversity. In each scatter plot, designs are included if at least one parameter is known. A short line is drawn in the direction of an unknown parameter. It centers on a value that would equal the most limiting factor (MLF) of the design, relative to the human eye.

Table 3 suggests that VF is one of two MLFs of the prototype. However, this is because, to conserve resources, a small array of pixels was designed. Using Equation (8), the VF would be 1.1 sr, or 37 dB better, for a 480 × 640 array of the same pixels. Although feasible, the power consumption would then be 1.55 W, or 40 dB worse, assuming it is proportional to the number of pixels. Consequently, array scaling alone cannot lead to a design that rivals the eye in these two parameters simultaneously. Power consumption must be reduced, especially for a megapixel array to become feasible.

The digital power of the proposed architecture, currently 72% of the total power, is expected to decrease significantly with CAS improvements and process scaling [18]. For example, readout circuits were not the focus when the DPS was designed. To read out a pixel bit serially, its shift register has to be cycled. At present, each time one pixel is read out, shift registers in all 3,071 other pixels are also cycled. During image sensing, parallel cycling contributes to efficiency. During readout, however, it is wasteful of digital power, which can be saved by adding a logic gate to each pixel.

Nevertheless, as shown in Figure 8a, the first prototype of the proposed architecture already has competitive power consumption among academic image sensors, which have comparable VF. Furthermore, commercial image sensors have 20–40 dB worse power consumption than the eye anyway, although their VF is getting competitive, especially with CCD technology.

Considering Table 3 and Figure 8b, SR and TR are not MLFs of the proposed architecture, but they need improving. Both are expected to benefit from CAS improvements and process scaling [18]. Moreover, in comparison to another DPS array (design 22), which uses linear sensors and pulse-width modulation [21], the prototype has comparable SR and superior TR.

In terms of CAS improvements, noise may be traded for area to benefit SR. For example, circuits may be placed beneath modulator capacitors, which reduces pixel size by about 10%. As for the 14.5 ms readout time, which limits TR, this is partly because all pixels are read out sequentially. Instead, a row of pixels may be read out in parallel to a high-speed memory bank, which is then read out quickly.

Process scaling is expected to benefit SR and TR primarily by its impact on the digital part of the pixel. Impact on the analog part of the pixel remains a subject for further research. The decimator and readout, which are 100% digital, comprise 46% of the total area. Digital circuits are likely to shrink with scaling. Because digital logic gets faster with scaling, readout time is likely to shorten.

Figure 8c shows PSNDR and/or PSNR *versus* DR. SNDR is a better measure of image quality than SNR [4]. Moreover, psychovisual experiments quantify SNDR, not SNR, of the human eye. However, image sensor documentation does not always provide the information required to compute PSNDR, and so, PSNR serves as an upper bound. Although commercial CCD arrays excel on PSNR, the prototype exceeds the human eye and rivals most other image sensors on PSNDR.

DR is best evaluated in the context of DL, its lower bound. Both are shown in Figure 8d. Because logarithmic pixels do not saturate easily (Figure 4), the prototype's DR undoubtedly exceeds the 110 dB shown (Figure 6) and probably exceeds the human eye's DR of 126 dB. What matters, however, is not to increase the BL of the test setup, but to decrease the DL of the architecture. Although the prototype's DL is its MLF (Table 3), the value is typical of other image sensors.

Skorka and Joseph [17] argue that vertical integration offers a path, compatible with the proposed architecture, to lower DL. They demonstrate a logarithmic VI-CMOS APS array, added to Figure 8 (design 26), that exhibits low DL and wide DR. SR was limited by lack of access to fine-pitch vertical integration, such as through-silicon vias. A hydrogenated amorphous-silicon photodetector array was flip-chip bonded onto a CMOS APS array, achieving 100% fill factor, *i.e.*, ratio of detector to pixel area. In contrast, the CMOS DPS array presented here (design 25) has a 2.3% fill factor.

Returning to Figure 8c, if the DL of the proposed architecture is decreased, or the BL of the test setup is increased, then the approach would simultaneously achieve, relative to the human eye, wide DR and high PSNDR. As shown, this has proven difficult. For example, designs 20, 21 and 26 are logarithmic or “lin-log” (design 21) APS arrays having chip, column and board-level ADCs, respectively. All suffer from low PSNDR. Whereas other research (designs 23 and 24) has successfully increased the DR of linear sensors via multiple sampling, this research (design 25) has successfully increased the PSNDR of logarithmic sensors via pixel-level ΔΣ ADCs.

## 5. Conclusions

Researchers are pursuing a variety of methods for high-performance CMOS image sensors. They can be categorized in many ways, such as linear and nonlinear sensors. Compared to the human eye, conventional linear sensors have high PSNDR, but narrow DR, whereas conventional logarithmic sensors have wide DR, but low PSNDR. To achieve a PSNDR that rivals the human eye, an important benchmark, this work has proposed a new architecture for logarithmic sensors, where each pixel includes a ΔΣ ADC, modulator and decimator.

A prototype of the proposed architecture, namely a logarithmic CMOS DPS array, was designed, built and tested, using a standard 0.18 μm CMOS process. Compared to other image sensors, SR is low due to large pixels. However, the approach has otherwise proven competitive. To increase SR, CAS improvements and process scaling may be employed, where the latter alone is expected to shrink the significant digital part of the pixel. Nevertheless, the SR is comparable, at present, to a linear CMOS DPS array published by independent researchers.

Signal and noise properties were specified at video rates, after a careful characterization using a commercial uniform source system. Compared to other image sensors from a recent survey, the prototype is distinguished by its simultaneous high PSNDR and wide DR. Although DR probably exceeded that of the human eye, what matters for most image sensors, including the prototype, is to reduce DL, and vertical integration is a compatible way to do so. Finally, the prototype demonstrated a PSNDR that exceeded that of the human eye, which has not been shown previously with logarithmic sensors.

## Figures and Tables

**Table 1.** Detailed schematics of the in-pixel ΔΣ analog-to-digital converters (ADCs). These were previously published in a refereed conference proceeding [14] and a US patent application [15], where the latter is freely available online.

**Detailed schematic**	[14]	[15]
Switched-capacitor modulator:	Figure 1	Figure 8
Operational transconductance amplifier	Figure 2	Figure 9
Common-mode feedback circuit		Figure 9
Modified regenerative-latch comparator	Figure 3	Figure 10
Bit-serial decimator:	Figure 4	Figure 3
Pulsed-latch D flip flop	Figure 5	Figure 4
Coefficient generator		Figure 5

**Table 2.** Specifications of the image sensor prototype. Due to lens geometry, 1 cd/m^2^ in the scene implies 0.4 lx on each pixel. Here, “SNR” and “SNDR” mean signal-to-noise ratio and signal-to-noise-and-distortion ratio, respectively.

**Parameter**	**Specification**
CMOS process	0.18 μm 1P6M
Array size	48 × 64
Pixel size	38 × 38 μm^2^
Detector size	5.8 × 5.8 μm^2^
Video rate	30 fps
Duty cycle	57%
Analog power	4.3 mW
Digital power	11.2 mW
Peak SNDR	45 dB
Peak SNR	46 dB
Dark limit	0.28 cd/m^2^
Dynamic range	> 110 dB

**Table 3.** Imaging performance relative to the human eye. Method and human eye data are from Skorka and Joseph [4].

**Parameter**	**Human eye**	**Prototype**	**Relative**
Power consumption	10 mW	15.5 mW	−4 dB
Visual field	4.1 sr	0.015 sr	−49 dB
Spatial resolution	50 cpd	3.9 cpd	−22 dB
Temporal resolution	65 Hz	15 Hz	−13 dB
Peak SNDR	36 dB	45 dB	+9 dB
Dark limit	0.001 cd/m^2^	0.28 cd/m^2^	−49 dB
Dynamic range	126 dB	> 110 dB	> −16 dB
